# Occupational accidents involving pesticides notified by SINAN

**DOI:** 10.47626/1679-4435-2022-864

**Published:** 2023-08-08

**Authors:** Luciane de Paula Machado, Jorge Mesquita Huet Machado, Bianca Coelho Moura

**Affiliations:** 1 Occupational Safety, Instituto Federal do Tocantins, Campus Palmas, Palmas, TO. Brazil; 2 Fundação Oswaldo Cruz, Medicine, Brasília, DF, Brazil

**Keywords:** occupational accident, pesticides, accident notification, acidente de trabalho, agrotóxicos, notificação de acidentes

## Abstract

**Introduction:**

The present article analyzed the number of pesticide accidents registered in Tocantins. In the case of occupational exposure to pesticides, the impact of pesticides on farmers is particularly great during direct handling or improper storage. This paper aims, through notifications of industrial accidents involving pesticides, to provide its target audience with information on notifications of accidents.

**Objectives:**

To analyze notifications of occupational accidents with pesticides via SINAN in the state of Tocantins, northern Brazil, from 2012 to 2017.

**Methods:**

The research deals with an explanatory literature that provides greater familiarity with the problem, making it clear or structured.

**Results:**

We can emphasize that pesticide poisoning is a serious public health problem. Among notifications of this event, 178 involved three types of pesticides, accounting for 12.35% of pesticide notifications. Furthermore, the most marketed pesticides are those whose formulation is based on the following active ingredients: glyphosate, 2,4-D, and mancozeb.

**Conclusions:**

After analyzing the data, we observed that the challenges brought by pesticide poisoning are related to the eligibility of the database of occupational accidents involving exogenous intoxications, being necessary to replace the use of agricultural pesticides, use pesticides safely for workers and the population, and seriously combat underreporting.

## Abstract (pt)

**Introdução:**

O presente artigo analisou o número de acidentes de agrotóxicos registrados no Tocantins. No caso de exposição ocupacional a pesticidas, o impacto dos pesticidas sobre os agricultores é particularmente grande durante o manuseio direto ou armazenamento inadequado. Este trabalho propõe, por meio das notificações de acidentes industriais envolvendo agrotóxicos, fornecer ao seu público-alvo informações sobre notificações de acidentes.

**Objetivos:**

Analisar as notificações de acidente de trabalho com agrotóxicos via SINAN no estado do Tocantins entre os anos de 2012 e 2017.

**Métodos:**

A pesquisa trata de uma literatura explicativa que proporciona uma maior familiaridade com o problema, tornando-o claro ou estruturado.

**Resultados:**

Podemos enfatizar que a intoxicação por agrotóxicos é um sério problema de saúde pública. Dentre as notificações desse evento, 178 envolveram três tipos de agrotóxicos, representando 12,35% das notificações de agrotóxicos. Os agrotóxicos mais comercializados foram aqueles com formulação à base dos seguintes ingredientes ativos: glifosato, 2,4-D e mancozebe.

**Conclusões:**

Após a análise dos dados, observamos que os desafios colocados pela intoxicação por agrotóxicos estão relacionados à elegibilidade do banco de dados de acidentes de trabalho envolvendo intoxicações exógenas, sendo necessário substituir o uso de defensivos agrícolas, usar agrotóxicos com segurança para o trabalhador e para a população, e combater seriamente as subnotificações.

## INTRODUCTION

Occupational exposure to pesticides during direct handling or resulting from improper storage and use of packages and clothes or from water pollution affects mainly farmers, causing accidents with these pesticides. The Article 19 of Law no. 8,213 on Social Security defines occupational accidents as those that occur when performing work in a company’s service or by the exercise of work by especially insured workers mentioned in item VII of article 11 of the aforementioned Law, covering situations that could cause body injury or functional disorder that leads to death or to the permanent or temporary loss or reduction of work capacity.^1^

Occupational accidents caused by pesticide poisoning correspond to intentional or unintentional poisoning due to ingestion, inhalation or skin absorption of chemical products during work activities or work-related journeys.^2^

Several terms can also be used to refer to pesticides, such as agricultural chemicals, agro chemicals, and poisons. These are some expressions related to a group of chemicals used to control animal and vegetable pests and plant diseases.^3^

Since Brazil is the world’s largest consumer of pesticides, the use of these substances has a significant impact on public health, covering a wide geographical area and involving different population groups, such as workers in several agricultural branches. Pesticides are one of the most important risk factors for the health of the general population, especially for workers, and for the environment.

According to the Federal Laws no. 7,802/89,^4^ and 4,074/02,^5^ which regulate the use of pesticides, these substances are defined as: “products and reactants of physical, chemical or biological processes, designed for use in the sectors of production, storage, and processing of agricultural products, as well as in pastures, protection of forests, either native or planted, other ecosystems, and urban, water, and industrial environments, whose purpose is to alter the composition of the flora or the fauna, in order to preserve them from the harmful action of living beings considered noxious.”

Notification is known to be a crucial tool for epidemiological surveillance, since it is the event from which the information-decision-action process is triggered. Notifications of pesticide poisoning to the Notifiable Diseases Information System (Sistema de Informação de Agravos de Notificação, SINAN) became compulsory after Ordinance no. 168 (National Health Surveillance Secretariat, Secretaria Nacional de Vigilância à Saúde/Ministry of Health, Ministério da Saúde [SVS/MS]), of May 05, 1997. Data collection for the present study was made using this system and included documents published from 2012 to 2017. The issue of occupational accidents provides a reasonable justification for this investigation.

This research is focused on the study of notifications of industrial accidents involving pesticides, to provide its target audience with useful information on accident notifications. The present study aimed to analyze notifications of occupational accidents with pesticides, using data available in SINAN. Furthermore, it presents the number of accidents notified in the state of Tocantins, northern Brazil, in order to assess the causes of these accidents and to classify safety measures to prevent accidents.

## METHODS

The aim of the present study was to conduct applied research, because it used basic research knowledge to solve problems. In order to better meet the objectives and to better understand this investigation, we decided to conduct exploratory research.

The following materials were used in the bibliographic survey: books, scientific articles, journals, and electronic documents published from 2012 to 2017, so as to conduct the search and allocation of knowledge on notified cases of occupational accidents with pesticides by analyzing data on the accidents notified on SINAN.

This investigation is considered a bibliographic study, having thus an explanatory nature, which in turn provides greater familiarity with the problem, by making it explicit or building hypotheses about it especially through a bibliographic survey.^6^

As a procedure, it is worth mentioning the need for a bibliographic survey, because we made use of previously published material, especially books. Furthermore, we understand that bibliographic survey is an important procedure of document research as a technical procedure.

This study aims to conduct research in the field of occupational accidents through a bibliographic survey. This is a study on the notifications of occupational accidents involving pesticides and a general analysis of data on accidents notified on SINAN.

Relevant data about accidents caused by pesticide poisoning were obtained from reports covering from 2012 to 2017 and were stratified by Health Regions, with the purpose of quickly analyzing the use of pesticides and their toxicology. The state of

Tocantins registered 154 confirmed cases of exogenous poisoning on SINAN from 2012 to 2017. Data were collected from the SINAN NET-Tocantins website, at the link: http://tabnet.datasus.gov.br/cgi/deftohtm.exe?sinannet/cnv/Intoxto.def, considering the following inclusion criteria: 1) Notifications by Health Region [Regional Interagency Commission, Comissão Intergestores Regional (CIR) of notification]; 2) Toxic agent: pesticide for agricultural use; 3) Exposure resulting from work: yes; 4) Final classification: confirmed poisoning.

**Figure 1:**
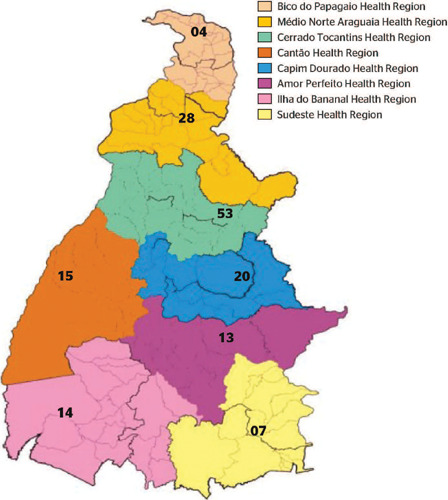
Notifications of confirmed cases of exogenous poisoning in Tocantins registered on the Notifiable Diseases Information System (Sistema de Informação de Agravos de Notificação, SINAN) from 2012 to 2017. Source: Adapted from the State Health Department of Tocantins.

The following map presents the number of notifications during the years studied in each Health Region (Figure 1).

## RESULTS AND DISCUSSION

According to data from the National Health Surveillance Agency (Agência Nacional de Vigilância Sanitária, ANVISA),^7^ Brazil is the world’s largest consumer of pesticides, accounting for 86% of pesticides used in Latin America. Of all pesticides consumed in Brazil, 58% are herbicides, 21% are insecticides, 12% are fungicides, 3% are acaricides, and 7% are other types of pesticides. In 2011, the states with the largest consumption of pesticides were São Paulo (346,079.2 t), Mato Grosso (132,478.3 t), Paraná (112,507.5 t), Minas Gerais (86,516.3 t), Rio Grande do Sul (71,772.9 t), Goiás (62,398.8 t), and Mato Grosso do Sul (50,609.7 t), whereas the states with the lowest consumption were Roraima (512.3 t), Amazonas (168.1 t), and Amapá (98.7 t).

Ordinance no. 1.271, of June 6, 2014 (which revoked Ordinance no. 104, of January 25, 2011), includes pesticide poisoning in the Compulsory Notification List and states that it should be notified using the exogenous poisoning form of the SINAN.^8^ Chart 1 shows the municipalities of the state of Tocantins with notified diseases grouped according to their respective Health Regions.

**Chart 1:** Municipalities with notified health diseases grouped according to their respective Health Region, Tocantins

Health Region	Municipalities with notified health diseases, January/April 2018
Bico do Papagaio	Ananás, Augustinópolis, Esperantina, Luzinópolis, Maurilândia do Tocantins, Nazaré, Palmeiras do Tocantins, Riachinho, Santa Terezinha do Tocantins, São Bento do Tocantins, São Sebastião do Tocantins, Tocantinópolis.
Médio Norte Araguaia	Aragominas, Araguaína, Araguanã, Babaçulândia, Barra do Ouro, Campus Lindos, Carmolândia, Darcinópolis, Goiatins, Murcilândia, Nova Olinda, Pau Darco, Piraquê, Santa Fé do Araguaia, Xambioá.
Cerrado Tocantins Araguaia	Arapema, Bandeirantes do Tocantins, Bernardo Sayão, Bom Jesus do Tocantins, Brasilândia do Tocantins, Centenário, Colinas do Tocantins, Colméia, Couto de Magalhães, Guaraí, Itacajá, Juarina, Palmeirante, Pedro Afonso, Pequizeiro, Presidente Kennedy, Recursolândia, Tupiratins.
Capim Dourado	Aparecida do Rio Negro, Fortaleza do Taboção, Lagoa do Tocantins, Lajeado, Miracema do Tocantins, Miranorte, Novo Acordo, Palmas, Rio dos Bois, Rio Sono, Santa Tereza do Tocantins.
Amor Perfeito	Brejinho de Nazaré, Chapada de Natividade, Fátima, Mateiros, Natividade, Pindorama do Tocantins, Ponte Alta do Tocantins, Porto Nacional, Santa Rosa do Tocantins, Silvanópolis.
Cantão	Araguacema, Barrolândia, Caseara, Cristalândia, Divinópolis do Tocantins, Dois Irmãos do Tocantins, Lagoa da Confusão, Paraíso do Tocantins, Pium, Pugmil.
Ilha do Bananal	Aliança do Tocantins, Alvorada, Araguaçu, Cariri do Tocantins, Crixás, Dueré, Figueirópolis, Formoso do Araguaia, Gurupi, Jaú do Tocantins, Palmeirópolis, Peixe, Sandolândia, São Salvador, São Valério da Natividade, Sucupira, Talismã.
Sudeste	Almas, Arraias, Aurora do Tocantins, Combinado, Conceição do Tocantins, Dianópolis, Novo Alegre, Paranã, Rio da Conceição, Taguatinga.

Source: Notifiable Diseases Information System (Sistema de Informação de Agravos de Notificação, SINAN).

The state of Tocantins has 139 municipalities, distributed into eight Health Regions, as shown in Chart 1.

According to data from the International Labor Organization, Brazil is the fourth leading country in the number of occupational accidents, after China, with approximately 15 million events, the United States, with 57 million, and Russia, with 3.1 million. Data from the European Union are not encouraging, since they only refer to formal workers with an employment contract and social security benefits.^9^

According to the World Health Organization,^10^ pesticides are estimated to cause 70,000 cases of acute and chronic diseases leading to death among workers from developing countries, and at least 7 million nonfatal acute and chronic diseases.

As shown by our research in the state of Tocantins, pesticide use entails high costs for both human and environmental health, and even to economic losses, due to the high number of accidents with pesticides. Table 1 shows report likelihood ratio (RLR), number, and incidence of cases of pesticide poisoning.

**Table 1:** Report likelihood ratio, number and incidence of cases of pesticide poisoning stratified by Health Region in the state of Tocantins from 2012 to 2017

Health Region	Municipalities (n)	Population* (b)	Cases (a)	Incidence rate† (= a/b)	RPR‡
Capim Dourado	14	367642	20 × 10^6^	5.44	0.55
Cantão	15	128,688	15 × 10^6^	11.66	1.18
Amor Perfeito	13	110,751	13 × 10^6^	11.74	1.19
Ilha do Bananal	18	183,258	14 × 10^6^	7.64	0.77
Sudeste	15	98,129	7 × 10^6^	7.12	0.72
South macroregion	75	888,468	60 × 10^6^	7.77	0.78
Cerrado Tocantins Araguaia	23	160,425	53 × 10^6^	33.04	3.34
Médio Norte Araguaia	17	298,152	28 × 10^6^	9.39	0.95
Bico do Papagaio	24	208,184	4 × 10^6^	1.92	0.19
North macroregion	64	666,761	85 × 10^6^	12.75	1.29
TOTAL	139	1,555,229	154	9.90 (B)

RLR = report likelihood ratio by region.

* According to the Brazilian Institute of Geography and Statistics (2018).

† a/b × 10^6^ (2012-2017).

‡ a/b ÷ B × l00.

Data from the table were analyzed using incidence rate = a/b × 10^6^ and RLR by region = a/b ÷ B × 100. It is possible to observe that, in the years studied, the

Cerrado Tocantins Araguaia Health Region, which includes 23 municipalities, had the highest likelihood report, with an RLR of 3.34.

As a strategy to reduce the number of accidents with pesticides, according to legislation, suspected and confirmed cases of exogenous pesticide poisoning are notified, as well as diseases related to occupational exposure to these substances, so as to promote opportunities for intervention. Ordinance MS/Minister’s Office (Gabinete do Ministro, MS/GM) no. 1,271, of June 2014, covers procedures for the compulsory notification of diseases, injuries, and public health events in public and private services throughout the national territory, providing opportunities for intervention. Another action is investigating all cases of exogenous pesticide poisoning to investigate their route of exposure and whether there are related or suspected cases of contact or poisoning.

One of the main problems and challenges of regulating the use and application of pesticides in Brazil is the lack of effective assessment of the toxicological risks of these substances for the population. It is important to promote comprehensive health protection and promotion actions, and to provide prevention and care services from a broad perspective of comprehensive medical care to people exposed to pesticides.^10^

**Figure 2:**
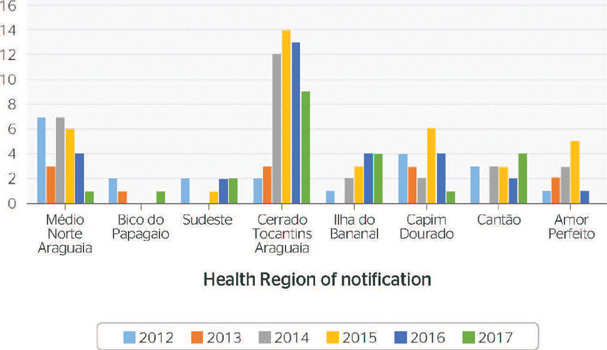
Number of notifications in the state of Tocantins from 2012 to 2017. CIR = Comissão Intergestores Regional.

Furthermore, there is the need to promote educational health actions and exchanges, as well as strengthening participation and social control to provide sustainability of actions and planned activities, in order to promote and monitor the health of professionals directly exposed to risks of accidents with pesticides in the state of Tocantins. Figure 2 presents the number of notifications in the state from 2012 to 2017.

The graph above shows the notifications distributed into Health Regions, demonstrating the number of accidents caused by pesticide poisoning in the state of Tocantins and indicating that the Cerrado do Tocantins Araguaia Health Region accounted for 34.41% of cases notified in the Health Regions.

The epidemiological study of pesticide poisoning in Brazil and in Tocantins is a field yet to be explored, due to the lack of available data and the need to use secondary research data. Furthermore, there are many difficulties to conduct the research. A problem worth highlighting is underreporting, since workers do not seek medical care when the first symptoms of poisoning appear.^8^

In 2017, there were 1,515 notifications of poisoning caused by all toxic substances in Tocantins. Of these, 178 notifications involved the three types of pesticides (for agricultural, household, and public health). There are 22 types of pesticides specifically related to agricultural activities in the aforementioned state, of which three account for 12.35% of notifications of pesticide poisoning, and the most marketed were those whose formula is based on the following active ingredients: glyphosate, 2,4-D, and mancozeb.^8^

It bears emphasizing that pesticide poisoning is a serious public health problem. In most cases, the increase of cases in urban areas is related to the abusive use of pesticide-based agricultural products, including rodenticides, which are illegally marketed under several names, such as aldicarb (commonly known as *chumbinho*), rodenticides, etc.

Among the measures to prevent pesticide poisoning, the most remarkable are educational actions on the use and the impact of pesticides on health and environment, as well as ways to prevent or minimize these impacts. Health monitoring practices directed at people exposed to pesticides include a series of comprehensive promotional actions for health prevention, protection, and maintenance, encompassing all entities affiliated to the Brazilian Unified Health System (regulatory agency), in addition to health and social institutions.^8^

The present research highlights that pesticides pose a significant risk for human health and the environment and should only be used under agronomic prescription and after performing all safety procedures. It is also worth mentioning that the number of accidents with pesticides increased by 25.66% in 2019 in the state of Tocantins compared to the previous year.^8^

Tocantins, one of the 27 Federative units of Brazil, was created by the 1988 Constitution, and its capital city is Palmas. This state has an area of 277,620.914 km^2^, comprises 139 municipalities, and has a population of 1,383,455 inhabitants.^10^ Furthermore, it has a strategic geographical position, which, together with the abundance of natural resources, especially water, with makes the state of Tocantins the ideal space for agricultural expansion.^11^

In 2015, the total area of planted crops was 1,173,302 hectares in the state of Tocantins, and the consumption of pesticides reached 17,403,387 liters, distributed into the following crops: soy (69%), corn (13%), rice (10%), cane (3%) watermelon (l%), and bean (1%).^11^

The most used pesticides in the state of Tocantins are Heroxinioa, Nomolt, Opera, Fox, Azimut, and Roundup for soy crops, Assist and Opera for corn crops, and Padron, Dominium, Platinum, Tordon, Truper, Defender, and Zartan for the maintenance of pastures. In 2014, Tocantins showed a rate of sales of pesticides and related products per planted area of 13.4 kg/ha.^11^

Despite the reduced number of notifications, it was observed that, in municipalities of Tocantins that had notified cases of poisoning related to agricultural cultures/crops, these cases were underreported and were not submitted to the monitoring system. This characterizes monocultures as spaces that reproduce the health impacts caused by pesticides, especially in the agribusiness.^11^

According to the Automatic Recovery System of the Brazilian Institute of Geography and Statistics (Sistema de Recuperação Automática do Instituto Brasileiro de Geografia e Estatística, SIDRA-IBGE),^12^ which made a survey on agricultural production and on the estimated pesticide use in the main municipalities of the state of Tocantins, the amount of pesticides used per hectare was 11 L/ha on average, and presented variations among the main crops: 20 L/ha for cotton; 10 L/ha for rice; 5 L/ha for cane; 5 L/ha for bean; 1 L/ha for manioc; 5 L/ha for corn; and 10 L/ha for soy.

Finally, the main symptoms reported by workers were lower limb tingling, epigastric pain, headache, dizziness, memory changes, and sleep disorders. Taking into consideration reference values for the general population, the most observed laboratory changes were increased alkaline phosphatase, hyperglobulinemia, and increased gamma-glutamyl transferase.^11^

Changes in the immune system, such as reduced proliferative activity of lymphocytes and changes in leukocyte phagocytic capacity, were found, suggesting the presence of immunosuppression and early metabolic aging.^12^ The study concluded that pesticide poisoning is a serious public health problem and that additional safety measures should be taken, in addition to increasing the number of campaigns related to use of agro chemicals and their effects.^11^

## CONCLUSIONS

After analyzing the data, we observed that challenges brought by pesticide poisoning are related to the eligibility of databases on occupational accidents involving exogenous poisoning, with the need to increase the number of notifications and combat underreporting, in addition to promoting safe use. This represents a great challenge for all social actors involved in the issue of pesticide poisoning, since the idea of a safe production, in which the work process should equally produce both products and health, has not been widely implemented yet.^12^

In view of the need to strengthen the control of pesticide sales, it is necessary to improve the effectiveness of inspection measures and to increase the demand for agronomic prescriptions to combat smuggling, making it difficult to acquire these substances and thus reducing the cases of pesticide poisoning.^13^

The present research did not find evidence of a positive correlation between high consumption of pesticides in the municipalities and high incidence of acute pesticide poisoning; therefore, a further study is needed to confirm this relationship. There is the need for an information system that ensures the quality of collection, storage, systematization, and provision of information that reflects population’s reality in the database.

Additional response actions are necessary, with emphasis on control and prevention measures to ensure proper use and exposure to agro chemicals, in order to reduce the number of cases of acute pesticide poisoning. These actions include training of health care professionals, improving health surveillance services, increasing public awareness, and strengthening the control of agrochemical sales.
